# Poly[μ_2_-aqua-(μ_3_-2,5-dichloro­benzene­sulfonato)sodium]

**DOI:** 10.1107/S1600536810018118

**Published:** 2010-05-22

**Authors:** Mohammad T. M. Al-Dajani, Hassan H. Abdallah, Nornisah Mohamed, Chin Sing Yeap, Hoong-Kun Fun

**Affiliations:** a School of Pharmaceutical Sciences, Universiti Sains Malaysia, 11800 USM, Penang, Malaysia; b School of Chemical Sciences, Universiti Sains Malaysia, 11800 USM, Penang, Malaysia; c X-ray Crystallography Unit, School of Physics, Universiti Sains Malaysia, 11800 USM, Penang, Malaysia

## Abstract

In the title compound, [Na(C_6_H_3_Cl_2_O_3_S)(H_2_O)]_*n*_, the Na^I^ ion is penta­coordinated by three dichloro­benzene­sulfonate anions and two water mol­ecules, forming a distorted trigonal-bipyramidal geometry. The Na^I^ ions are bridged by the sulfonate groups and the water mol­ecules, leading to a polymeric layer structure parallel to the *bc* plane in which O—H⋯O hydrogen bonds are observed.

## Related literature

For general background to organic sulfonyl chloride compounds, see: Adams & Marvel (1941 ▶); D’Souza *et al.* (2008 ▶); Henze & Artman (1957 ▶); Uchiro & Kobayashi (1999 ▶). For the stability of the temperature controller used for the data collection, see: Cosier & Glazer (1986 ▶).
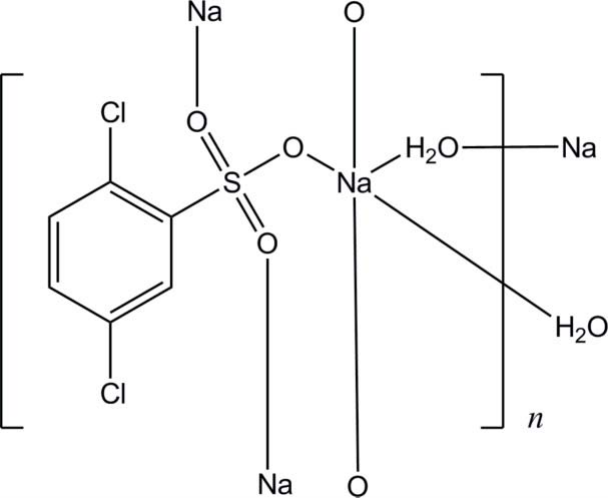


## Experimental

### 

#### Crystal data

[Na(C_6_H_3_Cl_2_O_3_S)(H_2_O)]
*M* *_r_* = 267.05
Monoclinic, 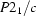

*a* = 17.2461 (10) Å
*b* = 5.4568 (3) Å
*c* = 10.7178 (6) Å
β = 106.190 (2)°
*V* = 968.64 (9) Å^3^
*Z* = 4
Mo *K*α radiation
μ = 0.91 mm^−1^
*T* = 100 K
0.34 × 0.34 × 0.05 mm

#### Data collection

Bruker APEXII DUO CCD area-detector diffractometer
Absorption correction: multi-scan (*SADABS*; Bruker, 2009 ▶) *T*_min_ = 0.749, *T*_max_ = 0.955
15240 measured reflections
4266 independent reflections
3594 reflections with *I* > 2σ(*I*)
*R*_int_ = 0.034

#### Refinement

*R*[*F* ^2^ > 2σ(*F* ^2^)] = 0.031
*wR*(*F* ^2^) = 0.100
*S* = 1.12
4266 reflections
127 parameters
H-atom parameters constrained
Δρ_max_ = 0.77 e Å^−3^
Δρ_min_ = −0.68 e Å^−3^

### 

Data collection: *APEX2* (Bruker, 2009 ▶); cell refinement: *SAINT* (Bruker, 2009 ▶); data reduction: *SAINT*; program(s) used to solve structure: *SHELXTL* (Sheldrick, 2008 ▶); program(s) used to refine structure: *SHELXTL*; molecular graphics: *SHELXTL*; software used to prepare material for publication: *SHELXTL* and *PLATON* (Spek, 2009 ▶).

## Supplementary Material

Crystal structure: contains datablocks global, I. DOI: 10.1107/S1600536810018118/is2546sup1.cif

Structure factors: contains datablocks I. DOI: 10.1107/S1600536810018118/is2546Isup2.hkl

Additional supplementary materials:  crystallographic information; 3D view; checkCIF report

## Figures and Tables

**Table 1:** Hydrogen-bond geometry (Å, °)

*D*—H⋯*A*	*D*—H	H⋯*A*	*D*⋯*A*	*D*—H⋯*A*
O1*W*—H1*W*1⋯O3^i^	0.75	2.09	2.8409 (13)	172
O1*W*—H2*W*1⋯O2^ii^	0.78	2.12	2.8620 (14)	162

Symmetry codes: (i) 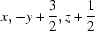; (ii) 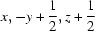.

**Fig. 1.**
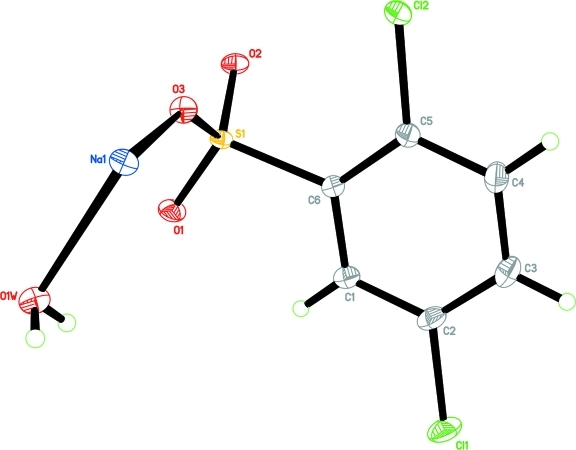
The asymmetric unit of the title compound with atom labels and 50% probability ellipsoids for non-H atoms.

**Fig. 2.**
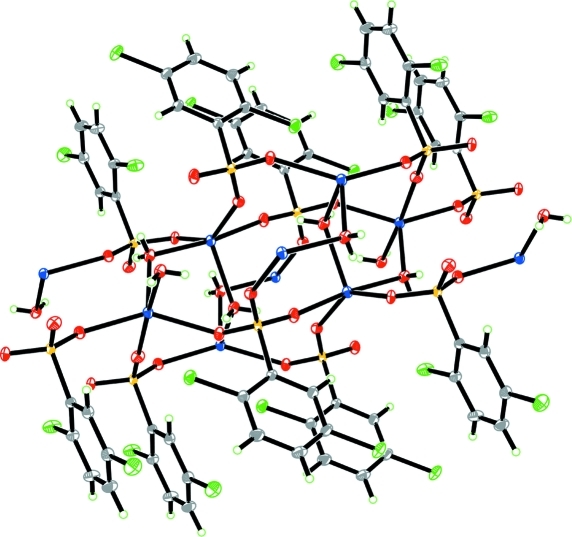
The molecular structure of the title compound with 50% probability ellipsoids for non-H atoms, showing the coordination environment for the NaI ion.

**Fig. 3.**
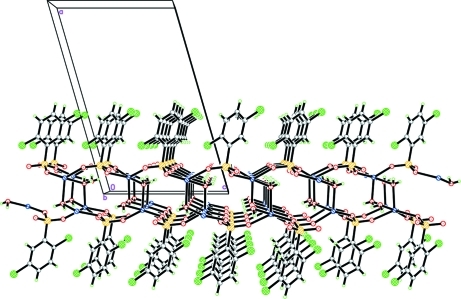
The crystal packing of title compound, viewed down the b axis, showing a polymeric plane parallel to the bc plane.

## supplementary crystallographic information

### Comment

Organic sulfonyl chloride compounds can be used as fundamental starting material for the synthesis of a variety of useful agricultural and medical compounds. They are widespread in many natural products and widely used as various artificial chemicals. It can be used as precursors in the synthesis of sulfonamide-based drugs (Adams & Marvel, 1941; D'Souza *et al.*, 2008; Henze & Artman, 1957; Uchiro & Kobayashi, 1999).

The asymmetric unit of the title compound contains one dichlorobenzenesulfonate anion, one sodium cation and one water molecule (Fig. 1). Each sodium cation is pentacoordinated with three dichlorobenzenesulfonate anions and two water molecules to form a distorted trigonal bipyramidal geometry (Fig. 2). In the crystal structure (Fig. 3), the molecules are linked into polymeric planes parallel to the *bc* plane. The polymeric structures are stabilized by the O1W—H1W1···O3 and O1W—H2W1···O2 hydrogen bonds (Table 1).

### Experimental

2,5-Dichlorobenzenesulfonyl chloride (0.02 mol, 4.86 g) was dissolved in 25 ml of 1,4-dioxane (C_4_H_8_O_2_) in round bottom flask with stirring. Sodium hydroxide (0.01 mol, 0.4 g) was added to the mixture and refluxed for 2 hours. The colour of the mixture was changed from colorless to light brown. After solvent evaporation, 50 ml of distilled water was added and mixed with 50 ml of butanol. After shaking the mixture for 15 min, butanol layer was isolated and brown precipitate was left after the butanol evaporation. The precipitate was dissolved in methanol at room temperature and left over night. The colourless plate crystals were formed, filtrated, washed with water and dried at 333 K.

### Refinement

Atoms H1W1 and H2W1 were located in a difference Fourier map and refined as riding on their parent atom, with *U*_iso_(H) = 1.5*U*_eq_(O). The remaining H atoms were positioned geometrically (C—H = 0.93 Å) and refined using a riding model, with *U*_iso_(H) = 1.2*U*_eq_(C).

### Crystal data

**Table:**

[Na(C_6_H_3_Cl_2_O_3_S)(H_2_O)]	*F*(000) = 536
*M**_r_* = 267.05	*D*_x_ = 1.831 Mg m^−^^3^
Monoclinic, *P*2_1_/*c*	Mo *K*α radiation, λ = 0.71073 Å
Hall symbol: -P 2ybc	Cell parameters from 5321 reflections
*a* = 17.2461 (10) Å	θ = 3.7–34.9°
*b* = 5.4568 (3) Å	µ = 0.91 mm^−^^1^
*c* = 10.7178 (6) Å	*T* = 100 K
β = 106.190 (2)°	Plate, colourless
*V* = 968.64 (9) Å^3^	0.34 × 0.34 × 0.05 mm
*Z* = 4	

### Data collection

**Table:**

Bruker APEXII DUO CCD area-detector diffractometer	4266 independent reflections
Radiation source: fine-focus sealed tube	3594 reflections with *I* > 2σ(*I*)
graphite	*R*_int_ = 0.034
φ and ω scans	θ_max_ = 35.1°, θ_min_ = 2.5°
Absorption correction: multi-scan (*SADABS*; Bruker, 2009)	*h* = −27→26
*T*_min_ = 0.749, *T*_max_ = 0.955	*k* = −8→7
15240 measured reflections	*l* = −17→17

### Refinement

**Table:**

Refinement on *F*^2^	Primary atom site location: structure-invariant direct methods
Least-squares matrix: full	Secondary atom site location: difference Fourier map
*R*[*F*^2^ > 2σ(*F*^2^)] = 0.031	Hydrogen site location: inferred from neighbouring sites
*wR*(*F*^2^) = 0.100	H-atom parameters constrained
*S* = 1.12	*w* = 1/[σ^2^(*F*_o_^2^) + (0.0518*P*)^2^ + 0.1259*P*] where *P* = (*F*_o_^2^ + 2*F*_c_^2^)/3
4266 reflections	(Δ/σ)_max_ = 0.001
127 parameters	Δρ_max_ = 0.77 e Å^−^^3^
0 restraints	Δρ_min_ = −0.68 e Å^−^^3^

### Special details

**Table:**

Experimental. The crystal was placed in the cold stream of an Oxford Cryosystems Cobra open-flow nitrogen cryostat (Cosier & Glazer, 1986) operating at 100.0 (1) K.
Geometry. All esds (except the esd in the dihedral angle between two l.s. planes) are estimated using the full covariance matrix. The cell esds are taken into account individually in the estimation of esds in distances, angles and torsion angles; correlations between esds in cell parameters are only used when they are defined by crystal symmetry. An approximate (isotropic) treatment of cell esds is used for estimating esds involving l.s. planes.
Refinement. Refinement of *F*^2^ against ALL reflections. The weighted *R*-factor wR and goodness of fit *S* are based on *F*^2^, conventional *R*-factors *R* are based on *F*, with *F* set to zero for negative *F*^2^. The threshold expression of *F*^2^ > σ(*F*^2^) is used only for calculating *R*-factors(gt) etc. and is not relevant to the choice of reflections for refinement. *R*-factors based on *F*^2^ are statistically about twice as large as those based on *F*, and *R*- factors based on ALL data will be even larger.

### Fractional atomic coordinates and isotropic or equivalent isotropic displacement parameters (Å^2^)

**Table:**

	*x*	*y*	*z*	*U*_iso_*/*U*_eq_	
Na1	0.08149 (3)	0.90539 (10)	0.72118 (5)	0.01189 (11)	
S1	0.148184 (17)	0.41852 (5)	0.54858 (3)	0.00956 (7)	
Cl1	0.42170 (2)	0.19246 (8)	0.92304 (3)	0.02371 (9)	
Cl2	0.25118 (2)	0.84404 (7)	0.45028 (3)	0.02048 (8)	
O1	0.12791 (6)	0.21059 (18)	0.61606 (9)	0.01572 (18)	
O2	0.13814 (6)	0.37277 (18)	0.41079 (8)	0.01294 (16)	
O3	0.10928 (6)	0.64583 (17)	0.57044 (8)	0.01330 (17)	
C1	0.29337 (8)	0.3214 (2)	0.72508 (11)	0.0135 (2)	
H1A	0.2667	0.1916	0.7514	0.016*	
C2	0.37368 (8)	0.3708 (3)	0.78932 (12)	0.0157 (2)	
C3	0.41525 (8)	0.5610 (3)	0.75190 (13)	0.0191 (3)	
H3A	0.4688	0.5921	0.7967	0.023*	
C4	0.37588 (8)	0.7047 (3)	0.64672 (14)	0.0191 (2)	
H4A	0.4032	0.8323	0.6199	0.023*	
C5	0.29559 (8)	0.6583 (2)	0.58129 (12)	0.0139 (2)	
C6	0.25339 (7)	0.4687 (2)	0.62083 (11)	0.01083 (19)	
O1W	0.04880 (6)	0.57236 (17)	0.84097 (9)	0.01383 (17)	
H1W1	0.0610	0.6420	0.9041	0.021*	
H2W1	0.0771	0.4600	0.8467	0.021*	

### Atomic displacement parameters (Å^2^)

**Table:**

	*U*^11^	*U*^22^	*U*^33^	*U*^12^	*U*^13^	*U*^23^
Na1	0.0144 (2)	0.0106 (2)	0.0110 (2)	−0.00028 (18)	0.00418 (17)	−0.00018 (16)
S1	0.01175 (13)	0.00815 (12)	0.00837 (11)	−0.00060 (9)	0.00211 (9)	0.00033 (8)
Cl1	0.01946 (16)	0.02930 (19)	0.01790 (14)	0.00684 (13)	−0.00218 (11)	0.00674 (12)
Cl2	0.01613 (15)	0.02089 (16)	0.02309 (15)	−0.00208 (11)	0.00327 (11)	0.01191 (12)
O1	0.0187 (4)	0.0124 (4)	0.0152 (4)	−0.0036 (3)	0.0034 (3)	0.0044 (3)
O2	0.0173 (4)	0.0120 (4)	0.0083 (3)	0.0001 (3)	0.0017 (3)	−0.0012 (3)
O3	0.0148 (4)	0.0117 (4)	0.0137 (4)	0.0018 (3)	0.0045 (3)	−0.0010 (3)
C1	0.0146 (5)	0.0136 (5)	0.0118 (4)	0.0021 (4)	0.0025 (4)	0.0005 (4)
C2	0.0143 (5)	0.0184 (6)	0.0127 (5)	0.0052 (4)	0.0009 (4)	0.0007 (4)
C3	0.0112 (5)	0.0232 (7)	0.0209 (6)	0.0010 (5)	0.0010 (4)	−0.0002 (5)
C4	0.0128 (5)	0.0203 (6)	0.0233 (6)	−0.0025 (5)	0.0038 (5)	0.0029 (5)
C5	0.0131 (5)	0.0136 (5)	0.0148 (5)	0.0000 (4)	0.0037 (4)	0.0025 (4)
C6	0.0113 (5)	0.0104 (5)	0.0105 (4)	0.0004 (4)	0.0027 (4)	−0.0003 (3)
O1W	0.0159 (4)	0.0109 (4)	0.0133 (4)	0.0000 (3)	0.0019 (3)	−0.0002 (3)

### Geometric parameters (Å, °)

**Table:**

Na1—O1^i^	2.2775 (10)	C1—C2	1.3905 (18)
Na1—O3	2.2974 (10)	C1—C6	1.3930 (17)
Na1—O2^ii^	2.3329 (10)	C1—H1A	0.9300
Na1—O1W^iii^	2.3427 (11)	C2—C3	1.383 (2)
Na1—O1W	2.3816 (11)	C3—C4	1.386 (2)
S1—O1	1.4400 (10)	C3—H3A	0.9300
S1—O2	1.4597 (9)	C4—C5	1.3900 (19)
S1—O3	1.4599 (10)	C4—H4A	0.9300
S1—C6	1.7841 (12)	C5—C6	1.3971 (17)
Cl1—C2	1.7393 (13)	O1W—H1W1	0.7531
Cl2—C5	1.7279 (13)	O1W—H2W1	0.7754
			
O1^i^—Na1—O3	86.09 (4)	C2—C1—C6	119.15 (12)
O1^i^—Na1—O2^ii^	86.09 (4)	C2—C1—H1A	120.4
O3—Na1—O2^ii^	144.45 (4)	C6—C1—H1A	120.4
O1^i^—Na1—O1W^iii^	90.95 (4)	C3—C2—C1	121.83 (12)
O3—Na1—O1W^iii^	114.30 (4)	C3—C2—Cl1	119.52 (10)
O2^ii^—Na1—O1W^iii^	100.45 (4)	C1—C2—Cl1	118.63 (11)
O1^i^—Na1—O1W	173.38 (4)	C2—C3—C4	118.99 (12)
O3—Na1—O1W	92.05 (4)	C2—C3—H3A	120.5
O2^ii^—Na1—O1W	91.77 (4)	C4—C3—H3A	120.5
O1W^iii^—Na1—O1W	95.60 (3)	C3—C4—C5	120.04 (13)
O1^i^—Na1—H1W1	160.3	C3—C4—H4A	120.0
O3—Na1—H1W1	107.2	C5—C4—H4A	120.0
O2^ii^—Na1—H1W1	74.6	C4—C5—C6	120.79 (12)
O1W^iii^—Na1—H1W1	96.4	C4—C5—Cl2	117.17 (10)
O1W—Na1—H1W1	17.3	C6—C5—Cl2	122.04 (10)
O1—S1—O2	113.36 (6)	C1—C6—C5	119.18 (11)
O1—S1—O3	113.71 (6)	C1—C6—S1	118.40 (9)
O2—S1—O3	112.18 (5)	C5—C6—S1	122.32 (9)
O1—S1—C6	105.24 (6)	Na1^vi^—O1W—Na1	119.73 (4)
O2—S1—C6	106.54 (5)	Na1^vi^—O1W—H1W1	117.2
O3—S1—C6	104.91 (6)	Na1—O1W—H1W1	92.9
S1—O1—Na1^iv^	173.06 (7)	Na1^vi^—O1W—H2W1	104.3
S1—O2—Na1^v^	134.05 (6)	Na1—O1W—H2W1	114.1
S1—O3—Na1	146.31 (6)	H1W1—O1W—H2W1	108.4
			
O1—S1—O2—Na1^v^	135.33 (8)	C3—C4—C5—Cl2	−179.44 (11)
O3—S1—O2—Na1^v^	4.87 (10)	C2—C1—C6—C5	1.66 (18)
C6—S1—O2—Na1^v^	−109.39 (8)	C2—C1—C6—S1	−174.79 (9)
O1—S1—O3—Na1	49.56 (13)	C4—C5—C6—C1	−1.65 (19)
O2—S1—O3—Na1	179.85 (10)	Cl2—C5—C6—C1	178.27 (10)
C6—S1—O3—Na1	−64.89 (12)	C4—C5—C6—S1	174.65 (10)
O1^i^—Na1—O3—S1	130.29 (11)	Cl2—C5—C6—S1	−5.43 (16)
O2^ii^—Na1—O3—S1	52.59 (14)	O1—S1—C6—C1	−3.24 (11)
O1W^iii^—Na1—O3—S1	−140.43 (10)	O2—S1—C6—C1	−123.88 (10)
O1W—Na1—O3—S1	−43.34 (11)	O3—S1—C6—C1	117.01 (10)
C6—C1—C2—C3	−0.5 (2)	O1—S1—C6—C5	−179.57 (10)
C6—C1—C2—Cl1	177.65 (9)	O2—S1—C6—C5	59.79 (12)
C1—C2—C3—C4	−0.6 (2)	O3—S1—C6—C5	−59.32 (11)
Cl1—C2—C3—C4	−178.80 (11)	O3—Na1—O1W—Na1^vi^	−84.32 (5)
C2—C3—C4—C5	0.6 (2)	O2^ii^—Na1—O1W—Na1^vi^	131.02 (5)
C3—C4—C5—C6	0.5 (2)	O1W^iii^—Na1—O1W—Na1^vi^	30.34 (5)

Symmetry codes: (i) *x*, *y*+1, *z*; (ii) *x*, −*y*+3/2, *z*+1/2; (iii) −*x*, *y*+1/2, −*z*+3/2; (iv) *x*, *y*−1, *z*; (v) *x*, −*y*+3/2, *z*−1/2; (vi) −*x*, *y*−1/2, −*z*+3/2.

### Hydrogen-bond geometry (Å, °)

**Table:**

*D*—H···*A*	*D*—H	H···*A*	*D*···*A*	*D*—H···*A*
O1W—H1W1···O3^ii^	0.75	2.09	2.8409 (13)	172
O1W—H2W1···O2^vii^	0.78	2.12	2.8620 (14)	162

Symmetry codes: (ii) *x*, −*y*+3/2, *z*+1/2; (vii) *x*, −*y*+1/2, *z*+1/2.
